# Disordered eating behaviors in youths with type 1 diabetes during COVID-19 lockdown: an exploratory study

**DOI:** 10.1186/s40337-020-00353-w

**Published:** 2020-12-02

**Authors:** Alda Troncone, Antonietta Chianese, Angela Zanfardino, Crescenzo Cascella, Alessia Piscopo, Anna Borriello, Serena Rollato, Francesca Casaburo, Veronica Testa, Dario Iafusco

**Affiliations:** 1 University of Campania “Luigi Vanvitelli”, Caserta, Italy; 2 Department of Psychology, University of Campania “Luigi Vanvitelli”, Viale Ellittico 31, Caserta, Italy; 3 Department of the Woman, of the Child and of the General and Specialized Surgery, Napoli, Italy

**Keywords:** Type 1 diabetes, Children, Adolescents, Disordered eating behaviors, COVID-19

## Abstract

**Background:**

Recent research indicates that patients with type 1 diabetes (T1D) are at higher risk for disordered eating behaviors (DEBs) than their peers without diabetes. The present study aimed to explore the prevalence of DEBs in a sample of Italian children and adolescents with T1D and in matched-pair healthy controls during the COVID-19 lockdown.

**Methods:**

In a cross-sectional study, 138 children and adolescents with T1D (aged 8.01–19.11 years, 65 boys) attending a Southern Italian diabetic service and 276 age- and gender-matched healthy peers voluntarily completed an online survey about eating behaviors (ChEAT and EAT-26), anthropometric characteristics, and clinical characteristics.

**Results:**

8.69% (*N* = 12) of participants with T1D and 13.4% (*N* = 37) of controls had ChEAT/EAT-26 scores indicating presence of DEBs, with no differences between patients—whether children (total ChEAT score F(1, 157) = .104, *p* = .748) or adolescents (total EAT-26 score F(1, 255) = .135, *p* = .731)—and healthy peers. zBMI values were lower than those measured in the latest diabetes visit (*p* < .0001), while HbA1c values remained unchanged (*p* = .110). In both groups, adolescents had lower Oral Control scores than children (T1D: F(1, 138) = 20.411, *p* < .0001, η^2^ = .132, controls: F(1, 276) = 18.271, *p* < .0001, η^2^ = .063); additionally, gender (female) and age were found to be significant predictors of several ChEAT/EAT-26 scores.

**Conclusions:**

This exploratory study suggested that children and adolescents with T1D did not experience more DEB symptoms during the COVID-19 lockdown compared to healthy controls. Results revealed DEBs as more of a female adolescent developmental issue rather than a result of the challenges of living with a chronic illness under quarantine measures. Possible effects of parental pressure on their children’s eating behaviors in the context of home confinement and of using a non-diabetes-specific measure to assess DEBs are discussed.

## Plain English summary

Living with T1D requires adherence to a demanding regimen entailing major lifestyle changes such as multiple daily insulin administrations (no longer produced by the pancreas), frequent blood glucose monitoring, specific diet and exercise programs. It is no surprise that people with T1D are considered as having an increased risk for psychological difficulties, such as behavioral disorders, anxiety symptoms, psychological distress and disordered eating behaviors. During the COVID-19 pandemic, the psychological condition of individuals suffering from T1D deserves special attention. This study aimed to explore the presence of disordered eating behaviors among Italian youth during home confinement, comparing a sample of individuals with type 1 diabetes to healthy control participants. Analysis of survey data indicated a lack of significant differences between these two groups, suggesting that youths with diabetes do not appear to be at greater risk for disordered eating during quarantine compared to their peers without T1D.

Overall, the data represent an important examination of eating behavior in a pediatric population during the COVID-19 pandemic and, given the known severe diabetes-related complications that result from disordered eating behavior, they are also encouraging in that differences were largely not observed between youth with and without T1D.

## Background

In response to the coronavirus disease 2019 (COVID-19) outbreak and to contain the spread of the infection, a temporary lockdown was announced by the Italian Prime Minister on March 9, 2020. Complete restriction on all international and domestic travel, social isolation, a nationwide school closure, and suspension of all non-essential services were established until May 3.

Quarantine has often been associated with several negative emotional consequences, decline in work performance, poor concentration, confusion, numbness, grief, insomnia, low mood that can reach depressive symptoms, psychological distress, and post traumatic/acute stress symptoms, along with long-term results such as alcohol abuse, dependency symptoms, and avoidance behaviors [1, 2].

During the time of the COVID-19 epidemic, studies conducted in China suggested that Chinese people experienced significant psychological distress [3]. During the level I emergency, moderate and high levels of psychological symptoms were observed in more than 70% of regular citizens [4]. Chinese people who were examined before and after the declaration of COVID-19 also showed more negative emotions (i.e., depression, anxiety, and indignation), fewer positive emotions, and less life satisfaction [5]. The outbreak was determined to affect the mental health of Chinese college students too, who consequentially showed symptoms of anxiety [6].

During the COVID-19 pandemic, the psychological condition of individuals suffering from type 1 diabetes (T1D)—who were already described by researchers as individuals needing particular attention [7–10]—can be critical for several reasons.

First, individuals with T1D might be more strongly influenced by the emotional responses related to COVID-19 as they suffer from a chronic disease. According to data from the general population in China, those with poor self-rated health status and a history of chronic illnesses experienced a significant psychological impact from the outbreak along with higher levels of stress, anxiety, and depression [11].

Second, people with T1D specifically are considered to be at an increased risk in general for psychological difficulties, such as behavioral disorders, anxiety symptoms, and psychological distress [12–15]. They are also at risk for developing eating disorders (EDs) and disordered eating behaviors (DEBs)—i.e., mild to extreme dieting behavior, including caloric restriction, skipping meals, binge eating attacks, unhealthy behaviors for weight control, and/or use of insulin restriction for intentional calorie purging [16, 17]. These problematic behaviors are reported more in youths with T1D compared to healthy peers, with the prevalence rate of DEBs/EDs at about 39.3%/7% in adolescents with T1D and about 32.5%/2.8% in those without T1D, and they are significantly associated with poorer glycemic control [18–23]. It has been assumed that specific elements of diabetes and its treatment (i.e., dietary restrictions, recurring weight variation, focus and attention to the body, food preoccupation, continuous attention to food intake, meal planning, counting carbohydrates, etc.) may generally facilitate the development of DEBs [24, 25].

Third, infection-containment measures are particularly traumatizing for children and adolescents: prolonged school closure, the lack of outdoor activities and interaction with friends and classmates, fear of infection, boredom, frustration, and lack of personal space at home were found to have a significant negative effect on children’s physical and mental health [1, 26]. The interaction between major lifestyle changes and the psychosocial stress caused by home confinement could create a vicious cycle, further aggravating the detrimental effects of the quarantine on youths’ physical and mental health [11].

As a result, in the time of COVID-19 children and adolescents with T1D require particular attention, because their psychological conditions might be naturally aggravated during a pandemic.

To date, several studies have been conducted on the medical aspects of COVID-19 highlighting that the present outbreak can worsen the condition of those with pre-existing mental health conditions and of those who are already vulnerable to psychosocial stressors [27, 28]. However, little research—both in the general population and in high-risk groups for psychological symptoms (such as T1D youths)—has explored the psychological difficulties experienced during periods of home confinement.

Consequently, the present study aims to investigate the psychological condition of youths with T1D during the COVID-19 lockdown. Specifically, the study was designed to: (1) evaluate the presence of DEB symptoms in a sample of Italian children and adolescents with T1D and in a sample of matched-pair healthy controls; and (2) to analyze the relationship between DEBs and sociodemographic, anthropometric, and clinical diabetes-related factors. It should be expected that, with limited free space to exercise, limited resources to implement a healthy lifestyle, difficulties in obtaining physician’s guidance, the increase of free time, and the reduction of school demands, the need to be busy in pleasant activities would have the potential to increase the focus on food in youths with T1D and their use of it to mitigate subjective stress.

## Method

### Participants and study procedure

The participants were recruited from among the patients attending a pediatric diabetes center in Southern Italy from April 1–30, 2020. To be included in the study, patients had to satisfy the following inclusion criteria: aged 8–19 years; T1D diagnosed at least 1 year prior to study enrollment; at least 6 months of using intermittently scanned continuous glycemic monitoring (CGM) device (Abbott FreeStyle Libre® Glucose Monitoring System, which was chosen as one of the CGM systems most used by patients attending the service); and absence of any significant developmental, cognitive, psychological, or medical conditions. Of the 960 patients registered in the electronic medical records/database as T1D patients who have attended the center in the last 12 months, 751 were selected according to the inclusion criteria: aged 8–19 years and diagnosed at least 1 year ago or longer; 209 were excluded because they did not fit these criteria (*N* = 110: less than 1 year since the onset; *N* = 99: younger than 8). In a second analysis of the 751 selected patients, 446 were excluded due to meeting other exclusion criteria (*N* = 43 affected by other medical condition; *N* = 403 not classified as using a CGM device), and 305 were determined to be eligible.

The parents of these patients were contacted via phone in order to further screen for eligibility as well as to invite them to participate to the study. Investigators called up to 3 times if subjects were not reached. During the phone call, parents were given a brief explanation of the study’s purposes and were asked to confirm that their son/daughter was currently using the FreeStyle Libre sensor. Parents whose child was confirmed as eligible and who verbally agreed to participate in the study were asked to visualize and read the mean blood glucose values collected by the CGM in the 2- and 4-week periods (15 and 30 days) preceding the study. These data were made available by the CGM device on demand, either from the user’s mobile phone or directly from the system. When necessary, a brief explanation was provided to enable the parent to find such data. Then, parents were sent a text message that gave them access to an informed consent form and a brief survey asking about their child’s sociodemographic and clinical data. The text message also included a link for their children to complete a web-based questionnaire assessing DEB symptoms (Google form).

In a similar procedure to that described for youth with T1D, control participants were recruited among medical and non-medical friends of the research team in the same period and the same geographic area. All children with known physical or psychological handicaps (confirmed by parents during the phone call) were excluded from the control group. From the healthy controls, participants were selected who best matched to the clinical group for age and gender (two control participants for each T1D case).

The study was approved by the local ethics committee and was conducted according to the principles of the Helsinki Declaration II.

### Measures

#### Sociodemographic and clinical data

A brief survey—answered by parents after they completed the consent form—was designed ad hoc for the study to record participants’ demographic and clinical data, including age, gender, height, weight, and (absence of) significant medical or psychological conditions (all participants). Patients’ duration of illness and the HbA1c values of their latest clinical visit (dated back to April 2019–March 2020) were collected from the electronic medical records. Since evidence suggests that at least 14 days of CGM data provide a good estimation of HbA1c values [29], a current HbA1c values estimation (only for participants with T1D) was obtained from the CGM mean glucose values of the one-month period (the previous 4 weeks). Estimated HbA1c was calculated according to ADAG (A1C-derived average glucose) Study Group data [30].

#### Weight status

BMI was used as a measure of actual weight status. Given that this index varies based on age and gender in children and adolescents, the BMI z-score was calculated for each participant based on gender, age, weight, and height, according to the Center for Disease Control (CDC) growth curve tables [31].

#### DEBs

The Eating Attitudes Test-26 (EAT-26) is one of the most widely-used standardized self-report screening measures to assess symptoms of eating problems and eating disorder risk in general [32]. It is a 26-item abbreviated version of EAT [33], with items rated on a 6-point scale (always, very often, often, sometimes, rarely, and never). It includes three subscales: Dieting (e.g., “Particularly avoid food with a high carbohydrate content”); Bulimia and Food Preoccupation (“Have gone on eating binges where I feel that I may not be able to stop”); and Oral Control (“Avoid eating when I am hungry”). The total score is scored as the sum of all items.

As suggested by Garner et al. [32] and consistent with previous research [34, 35], the EAT-26 total score cutoff of ≥20 indicates greater presence of symptoms associated with eating problems, to a level warranting attention and further investigation.

To examine eating attitudes and behaviors among children a modification of the EAT-26 for children—the Children Eating Attitudes Test (ChEAT) [36, 37]—was used. ChEAT is essentially the EAT with simplified language. Like EAT-26, it is comprised of 26 items scored on a six-point Likert scale, with higher scores indicating greater severity; a score of 20 has been used as a cutoff to identify disturbed eaters [36].

Much evidence has shown that ChEAT [36–40] and EAT-26 [32, 41–43] are reliable and valid psychometric tools to internationally assess abnormal eating attitudes. For the present study, a validated Italian version both the EAT-26 [35] and ChEAT [40] were used. Specifically, EAT-26 was administered to adolescents older than 13 years, and ChEAT was administered to children aged 8–13 years.

### Statistical analysis

To assess the homogeneity of the scale, the Cronbach’s alpha (α) was computed. Chi-square testing was used to test frequencies between groups, and Student’s *t*-tests were used to compare the means of the sociodemographic and clinical variables between the two groups (i.e., patients and control). Two-way ANOVA was used to examine the main effects of gender and group (T1D vs. controls), as well as gender × group interaction on DEBs (ChEAT and EAT-26 scores).

Hierarchical multiple regression analyses were conducted to evaluate the relationship between DEB and variables of interest (age, gender, zBMI, duration of illness, glycemic control). The ChEAT/EAT-26 scores (total and subscales) were the dependent variable. To analyze whether this relationship interacted with illness, regression analyses were performed separately for participants with T1D and for controls. Tolerance values of > 0.1 were considered acceptable to exclude multicollinearity [44]. 

All analyses were carried out with the raw scores. Results were considered significant at alpha = 0.05 for a two-sided test. The statistical analysis was conducted with Statistical Package for the Social Sciences (SPSS) version 21.0 for Macintosh.

## Results

### Sample characteristics

Out of 305 parents of children with T1D who were approached, 153 were excluded (*N* = 26 could not be reached by phone due to incorrect phone numbers or lack of answers, *N* = 127 patients had not been using the FreeStyle Libre sensor). In addition, *N* = 7 parents were unwilling to participate, due to general worries that their children would undergo psychological evaluation, temporary family problems, or reluctance/difficulty in using mobile phones and web-based information; *N* = 7 children refused to participate, due to lack of interest or because they were completing homework or busy with other activities.

In terms of the healthy control sample, of 310 parents approached, *N* = 5 could not be reached (due to lack of answers), and *N* = 5 parents refused to participate (out of perplexity regarding the possibility of their children undergoing psychological evaluation). *N* = 2 were excluded because a second analysis revealed that they did not meet the inclusion criteria (i.e., they suffered from chronic illnesses). Selections were made from the *N* = 298 healthy participants enrolled in order to achieve the best matching control peers (for age and gender).

In the end, the study samples consisted of 138 children and adolescents with T1D (65 m, 73 f) and 276 healthy peers (112 m, 164f). The demographic and clinical information of children with T1D are shown in Table 1.

**Table 1:** Demographic and clinical data of participants with T1D and controls

	T1D *N* = 138	Healthy controls *N* = 276	
	M (SD)	M (SD)	*p*
Gender (*N*) (male/female)	65/73	112/164	.206
Age (years)	13.67(3.21) (range 8.01–19.11)	13.78 (3.01) (8–19.11)	.725
Diabetes duration (years)	5.98 (3.22)	–	–
**HbA1c (%)**			
Estimation 15 days	8.45 (1.44)^a^	–	–
Estimation 30 days	8.42 (1.33)^b^	–	–
Latest visit	8.24 (1.2)	–	–
**z-BMI**			
Current	.53 (1.01)^c^	.42 (.96)	.353
Latest visit	.89 (1.03)	–	–

Data are presented as mean values and standard deviations unless otherwise stated

Abbreviations: *T1D* type 1 diabetes, *N* number of subjects, *z-BMI* standardized body mass index

^a^Compared with HbA1c as measured in latest visit (estimation 15 days t(137) = − 1.723, *p* = .087)

^b^ Compared with HbA1c as measured in latest visit (estimation 30 days t(137) = − 1.609, *p* = .110)

^c^ Compared with zBMI as measured in latest visit (t (137) = 8.102, *p* < .0001)

No statistically significant differences were found between the children with T1D and the control group in terms of gender (*X*^2^ = 1.599, *p* = .206), age (t(412) = −.352, *p* = .725), or zBMI (t(412) = 1.110, *p* = .267).

In participants with T1D, the current mean zBMI of .53(1.01) was significantly lower than that measured at the latest visit (t (137) = 8.102, *p* < .0001), while mean HbA1c values (15/30 days estimation) of 8.42% (68 mmol/mol) did not differ from those measured at the latest visit (15 days t(137) = − 1.723, *p* = .087; 30 days t(137) = − 1.609, *p* = .110). Factorial ANOVA confirmed that compared to healthy controls, participants with T1D did not differ in zBMI values (F(1,414) = .869, *p* = .353). Additionally, factorial ANOVA revealed no significant main effect of gender (F(1,414) = 496, *p* = .482) or interaction (gender × disease) (F(1,414) = 1.018, *p* = .313) on zBMI values.

### DEBs in DT1 and controls

The mean score for each ChEAT/EAT-26 subscale by group can be seen in Table 2.

**Table 2:** Ch-EAT/EAT-26 Cronbach’s alpha coefficients, mean scores in total sample, children and adolescents with and without T1D. Frequency of DEBs as measured by Ch-EAT/EAT-26 in total sample, children and adolescents with and without T1D. Comparisons of means and frequencies on the basis of illness and age

		Total sample			Children (≤13y)			Adolescents (>13y)			T1D	Controls
		T1D *N* = 138	Controls *N* = 276	T1D vs. Ctrl	T1D *N* = 51	Controls *N* = 107	T1D vs. Ctrl	T1D *N* = 87	Controls *N* = 169	T1D vs. Ctrl	Children vs. Adolescents	Children vs. Adolescents
	α	M (SD)	M (SD)	*p*	M (SD)	M(SD)	*p*	M (SD)	M (SD)	*p*	*p*	*p*
Ch-EAT/EAT-26												
Score ≥ 20% (N)		8.69 (12)	13.4 (37)	.162	3.9 (2)	14.9 (16)	.056	11.49 (10)	12.4 (21)	.83	.128	.827
Dieting	.695/.868	6.38(5.53)	5.8(6.88)	.132	5.20(3.91)	4.87(5.19)	.394	7.07(6.21)	6.38(7.72)	.225	.061	.093
Oral control	.656/ .698	2.23(2.55)	2.67(3.59)	.358	3.45(2.89)	3.79(3.93)	.694	1.52(2.03)	1.96 (3.18)	.413	<.0001	<.0001
Bulimia food preocc.	.529/.787	1.14(1.86)	1.41(2.58)	.427	1.14(1.39)	1.21(1.96)	.840	1.14 (2.1)	1.54 (2.9)	.410	.913	.564
Total score	.696/.909	9.75(7.71)	9.88(10.79)	.636	9.78(5.27)	9.87(7.59)	.748	9.72(8.87)	9.89(12.41)	.731	.916	.815

Abbreviations: *T1D* type 1 diabetes, *Ctrl* control, *Bulimia food preocc*. Bulimia and Food Preoccupation

The Cronbach’s alpha reliability coefficient for the EAT-26 and the ChEAT (total scores) showed satisfactory levels (Table 2).

#### Total sample

According to the ChEAT/EAT-26 scores, 8.69% (*N* = 12) of participants with T1D and 13.4% (*N* = 37) of controls had values of 20 or more, indicating presence of DEBs. No significant differences in DEB frequency were seen between patients and healthy controls (*X*^2^ = .1956, *p* = .162), between children (*X*^2^ = 3.66, *p* = .055) and adolescents with T1D (*X*^2^ = .134, *p* = .714) compared to matched healthy peers, between children with T1D and adolescents with T1D (*X*^2^ = 2.322, *p* = .128), and between children and adolescents of control (*X*^2^ = .048, *p* = .827).

Two-way ANOVA (disease × gender) indicated that participants with T1D did not score differently from healthy controls in any ChEAT/EAT-26 scales (Dieting F(1,414) = 2.282, *p* = .132; Oral control F(1,414) = .848, *p* = .358; Bulimia and Food Preoccupation F(1,414) = .631, *p* = .427; Total score F(1,414) = .224, *p* = .636).

There were main effects of gender for all of the ChEAT/EAT-26 subscales (Dieting F(1,414) = 27.207, *p* = .000, η^2^ = .062; Oral Control F(1,414) = 3.987, *p* = .047, η^2^ = .010; Bulimia and Food Preoccupation F(1,414) = 11.002, *p* = .001, η^2^ = .026; Total score F(1,414) = 24.118, *p* < .0001, η^2^ = .056), indicating that girls had significantly higher ChEAT/EAT-26 scores than boys.

There was also an interaction between disease and gender for two ChEAT/EAT-26 subscales and the Total score (Dieting F(1,414) = 4.954, *p* = .027, η^2^ = .012; Oral Control F(1,414) = 3.963, *p* = .047, η^2^ = .010; Total score F(1,414) = 5.621, *p* = .018, η^2^ = .014), with healthy boys having lower EAT scores than other groups. No interaction effects were found for Bulimia scores (F(1,414) = .888, *p* = .347).

#### Children

In a comparison of children (≤13y) with T1D to matched healthy controls, no significant differences were found in the ChEAT Total score (F(1, 157) = .104, *p* = .748) or in subscale scores (Dieting F(1,157) = .732, *p* = .394; Oral Control F(1, 157) = .155, *p* = .694; Bulimia and Food Preoccupation F(1,157) = .041, *p* = .840).

A main effect of gender was found for the ChEAT Total score (F(1, 157) = 5.811, *p* = .017, η^2^ = .036) and the Dieting (F(1,157) = 6.532, *p* = .012, η^2^ = .041) subscale—with girls having higher scores than boys—but not for Oral control (F(1,157) = 1.902, *p* = .170) or Bulimia and Food Preoccupation (F(1,157) = .125, *p* = .725), for which the scores did not differ between boys and girls.

There was an interaction effect of disease × gender only for Dieting scores (F(1,414) = 4.356, *p* = .039, η^2^ = .028), indicating that healthy boys had the lowest ChEAT scores compared to other groups. No interaction effects were found for Oral Control (F(1,157) = .263, *p* = .609), Bulimia and Food Preoccupation (F(1,157) = .111, *p* = .740), and Total score (F(1,157) = 3.259, *p* = .073).

#### Adolescents

In a comparison of adolescents (>13y) with matched healthy controls, no significant differences were found in the EAT-26 Total score (F(1, 255) = .135, *p* = .731) or in its subscales (Dieting F(1,255) = 1.418, *p* = .225; Oral Control F(1, 255) = .674, *p* = .413; Bulimia and Food Preoccupation F(1,255) = .680, *p* = .410).

For all comparisons, ANOVA indicated a significant main effect of gender on the EAT-26 Total score and subscale scores (Total score F(1,255) = 18.421, *p* < .0001, η^2^ = .068; Dieting F(1, 255) = 21.157, *p* = .000, η^2^ = .077; Bulimia and Food preoccupation F(1,255) = 16.360, *p* < .0001, η^2^ = .061) except for the Oral Control subscale (F(1,255) = 2.382, *p* = .124).

Interaction effects of disease × gender were only found for the Oral Control subscale (F(1,255) = 5.703, *p* = .018, η^2^ = .022), indicating that healthy girls had the highest EAT-26 scores compared to other groups. No significant interaction (gender × disease) effects were found for Total score (F(1, 255) = 3.203, *p* = .075), Dieting F(1,255) = 2.180, *p* = .141), or Bulimia and Food Preoccupation (F(1, 255) = .939, *p* = .333) scores.

#### Children vs. adolescents

In a comparison of children with T1D and adolescents with T1D, no significant differences were found in the ChEAT Total score (F(1,138) = .011, *p* = .916) or for two subscales (Dieting F(1,138) = 3.569, *p* = .061; Bulimia and Food Preoccupation F(1,138) = .012, *p* = .913). A main effect of age was found for the Oral Control subscale (F(1,138) = 20.411, *p* < .0001, η^2^ = .132), indicating that adolescents had lower scores than children.

No main effect of gender and no interaction effects (gender × age) were found for the ChEAT/EAT-26 Total score (gender F(1, 137) = 2.497, *p* = .116; interaction F(1,137) = 1.139, *p* = .288) or for Dieting (gender F(1,137) = 2.854, *p* = .093; interaction F(1,137) = 1.693, *p* = .195), Oral control (gender F(1,138) = .070, *p* = .792; interaction F(1,138) = 1.013, *p* = .316), or Bulimia and Food Preoccupation (gender F(1,138) = 1.651, *p* = .201, interaction F(1,138) = 3.774, *p* = .054) scores.

In a comparison of healthy children with adolescents, no significant differences were found in the ChEAT/EAT-26 Total score (F(1, 276) = .055, *p* = .815) or in two subscales (Dieting F(1,276) = 2.845, *p* = .093; Bulimia and Food Preoccupation (F(1,276) = .334, *p* = .564). A main effect of age was found in the Oral Control subscale score (F(1, 276) = 18.271, *p* < .0001, η^2^ = .063), indicating that adolescents had lower scores than children.

A main effect of gender was found for Total score (F(1, 276) = 17.825, *p* < .000, η^2^ = .093), Dieting (F(1,276) = 32.362, *p* < .0001, η^2^ = .106), Oral Control (F(1,276) = 9.254, *p* = .003, η^2^ = .033), and Bulimia and Food Preoccupation (F(1,276) = 7.263, *p* = .007, η^2^ = .026), indicating that girls had higher scores than boys.

No interaction effects were found for the ChEAT/EAT-26 Total score (F(1,276) = 2.277, *p* = .132), Dieting (F(1,276) = 1.345, *p* = .247), or Oral Control (F(1,276) = .109, *p* = .741). An age × gender interaction was only found in Bulimia and Food Preoccupation (F(1,276) = 7.370, *p* = .007, η^2^ = .026), indicating that adolescent girls had the highest scores of all groups.

### Predictors of DEBsges

Table 3 presents the results of a hierarchical regression predicting DEBs (ChEAT/EAT-26 scores) in participants with T1D and in healthy controls.

**Table 3:** Summary of linear regression analyses of variables predicting DEBs (ChEAT/EAT-26 subscales and total scores) in participants with T1D and controls

	T1D (*N* = 138)					Controls (*N* = 276)				
Variables Ch-EAT/EAT-26	B	SE B	*β*	*p*	Collinearity tolerance	B	SE B	*β*	*p*	Collinearity tolerance
**Dieting**										
Step 1										
Age	.290	.144	.168	.046	.999	.325	.129	.142	.012	.991
Gender	1.882	.923	.170	.043	.999	.4.623	.789	.331	<.0001	.991
	R^2^ = .059			.017		R^2^ = .138			<.0001	
Step 2										
Age	.368	.152	.213	.017	.798	.302	.129	.132	.020	.982
Gender	2.156	.884	.195	.016	.974	4.764	.790	.341	<.0001	.981
z-BMI	1.562	.440	.283	.001	.977	.729	.404	.102	.073	.983
Duration of illness	−.311	.156	−.181	.048	.759					
HbA1c	−.515	.337	−.124	.129	.950					
	ΔR = .118			.001		ΔR = .010			<.0001	
Total R^2^		.177				.	.149			
**Oral Control**										
Step 1										
Age	−.285	.064	**−.359**	<.0001	.999	−.297	.069	−.249	<.0001	.991
Gender	.053	.410	.010	.898	.999	1.533	.422	.210	<.0001	.991
	R^2^ = .129			<.0001		R^2^ = .096			<.0001	
Step 2										
Age	−.259	.072	−.326	. < .0001	.798	−.271	.067	−.227	<.0001	.982
Gender	.095	.416	.019	.820	.974	1.367	.413	.187	.001	.981
z-BMI	−.289	.207	−.114	.166	.977	−.859	.211	−.230	<.0001	.983
Duration of illness	−.036	.073	−.045	.626	.759					
HbA1c	−.034	.159	−.018	.830	.950					
	ΔR = .016			.001		ΔR = .052			<.0001	
Total R^2^		.144					.148			
**Bulimia and food preoccupation**										
Step 1										
Age	.064	.049	.110	.197	.999	.070	.051	.082	.171	.991
Gender	.569	.314	.153	.072	.999	1.000	.311	.191	001	.991
	R^2^ = .036			.081		R^2^ = .046			.002	
Step 2										
Age	.093	.055	.160	.094	.798	.061	.051	.071	.234	.982
Gender						1.058	.311	.202	.001	.981
z-BMI	.001	.159	.001	.994	.977	.298	.159	.111	.063	.983
Duration of illness	−.068	.056	−.118	.229	.759					
HbA1c	−.007	.122	−.005	.061	.950					
	ΔR = .011			.258		ΔR = .012			.001	
Total R^2^		.048					.058			
**Total score**										
Step 1										
Age	.069	.204	.029	.737	.999	.097	.205	.027	.637	.991
Gender	2.504	1.307	.163	.058	.999	7.156	1.259	.326	<.0001	.991
	R^2^ = .028			.152		R^2^ = .109			<.0001	
Step 2										
Age	.202	.224	.084	.369	.798	.092	.207	.026	.657	.982
Gender	2.882	1.296	.187	.028	.974	7.189	1.267	.328	<.0001	.981
z-BMI	1.274	.646	.166	.050	.977	.168	.649	.015	.796	.983
Duration of illness	−.415	.228	−.173	.072	.759					
HbA1c	−.556	.495	−.096	.263	.950					
	ΔR = .061			.030		ΔR = .000			<.0001	
Total R^2^		.089					.109			

Abbreviations: *T1D* type 1 diabetes, *z-BMI* standardized body mass index, *HbA1c* glycated hemoglobins

In most cases, the hierarchical regression equations for T1D and control participants was significant (step 1 total score T1D: F(2,137) = 1.910, *p* = .152, controls: F(2,275) = 16.682, *p* < .0001; step 2 total score T1D: F(2,137) = 2.669, *p* = .030, controls: F(2,275) = 11.106, *p* < .0001), accounting for approximately 5.8–17.7 of the variance of the majority of ChEAT/EAT-26 subscales (except for Bulimia and Food Preoccupation in T1D scores—step 1 F(2,137) = 2.555, *p* = .081; step 2 F(2,137) = 1.323, *p* = .258). Gender (female) was found to be a significant predictor of all ChEAT/EAT-26 subscale scores in controls and of ChEAT/EAT-26 Dieting and Total score in T1D participants; age was associated with the Dieting and Oral Control subscales in both groups. zBMI significantly predicted T1D participants’ Dieting scores and control participants’ Oral Control scores, while duration of illness was found to predict Dieting score in T1D patients (Table 3).

## Discussion

This study was the first to evaluate the psychological conditions experienced by youths with T1D during quarantine approximately 1 month after the COVID-19 outbreak in Italy. In particular, it examined frequency levels of DEBs and related associated factors in a sample of children and adolescents with T1D compared with gender- and age-matched healthy control individuals.

In contrast with previous evidence, both from research literature reviews [18, 19, 22, 23] and from Italian samples [45–50], the present findings suggest that comparisons of T1D youths/controls do not indicate a significant difference in occurrence of DEBs. In fact, the frequency of critical scores indicating greater presence of DEB symptoms in younger children was higher in controls than in youths with T1D, although this difference was borderline significant. This contradicts the general assumption that “social distancing” and isolation—creating anxiety, sadness, anger, and perception/sense of loneliness—may have a negative psychological impact [1] and even exacerbate eating disorder risks [51], further compromising individuals with psychopathological and eating problems [27, 28, 52, 53]. These results appear to be more consistent with Wang et al.’s study [54] —which described no significant differences in mental health problems in quarantined youths (undergraduate students) compared to non-quarantined peers—as well as with evidence in T1D literature indicating that higher levels of disordered eating attitudes and behaviors are not reported in youths with T1D compared to healthy comparison peers [55–58].

However, it is possible that other explanations might account for the present findings.

First, results concerning the occurrence of DEBs should be viewed with special caution, and the validity of the tools adopted to identify unhealthy eating habits should be taken into account. It is important to note that the measures used here to assess DEBs (ChEAT and EAT-26) are widely recognized as valid DEB detection tools for the general population [32, 36–43], but they have not been designed for use in people with T1D. Measuring DEBs using a generic DEB tool may fail to identify or inaccurately assess certain diabetes-specific eating behaviors, such as insulin omission or reduction [59, 60].

It is worth noting that the abovementioned studies that did not find higher DEB rates in adolescents with T1D  [55–58] all employed measures that were not adapted for individuals with T1D (e.g., SCOFF; EDE-Q) and acknowledged that their results allow the potential for biased answers (due to the lack of items asking about insulin manipulation) among individuals with T1, resulting in a reduction of the estimates of DEBs. In addition, like Pursey et al. [22] in their recent review on tools used to identify DEBs and EDs in people with T1D, these authors highlighted that the wide variety of tools used to assess DEBs in people with T1D—especially those not adequately validated for use in people with T1D (such as ChEAT and EAT-26)—may contribute to inconsistencies in the clinical identification of DEBs. They also reflected on the possible role of the huge variation in methodological approaches and differences in study design as well as in the characteristics of the study populations (age at onset, SES level, insulin regimen, BMI) in explaining inconsistent data research in this area.

It is necessary to wonder the extent to which the limitations of ChEAT and EAT-26 (in terms of appropriateness and validity for use in individuals with T1D) may have played a role in underestimating the prevalence of DEBs among T1D patients, thereby hiding differences between participants with T1D and controls and yielding seemingly higher rates of DEB occurrence in younger controls.

Second, it could be hypothesized that the present findings could be also explained as a possible effect of parental pressure on their children’s eating behaviors. It could be argued that due to home confinement, parents may monitor their children’s behavior throughout the day, preventing unhealthy conduct and exhorting them to better meet diabetes rules. In other words, the lockdown may have reduced patients’ opportunities to adopt/engage in the unhealthy eating behaviors or weight control practices (e.g., consumption of large quantities of high-fat foods, skipping meals, taking less insulin) that have been frequently observed in teens with diabetes [61, 62]. Furthermore, it is reasonable to suppose that the lockdown-imposed self-isolation may have reduced/canceled activities and contexts typically linked to social situation with peers that usually challenge good diabetes management (e.g., perceiving social pressures to eat inappropriate foods, eating out with friends and seeing them eating and drinking what they want, etc.) and lead to unhealthy behaviors affecting glycemic control [63].

The potential increased parental emphasis on the importance of diabetes control, along with the related pressure to engage in healthy eating behaviors and to take control of their weight, seems to be confirmed by the zBMI values (which were found to be lower than those measured in the latest diabetes visit) and by the HbA1c values remaining unchanged. It is worth noting that glycemic control—even though higher than the American Diabetes Association’s recommended target value of 7.5% for good metabolic control [64]—is substantially analogous to population data in similar age groups [65, 66]. It is also true that the differences in the methods of data collection at the latest visit (i.e., zBMI determined by clinically-measured height and weight data, HbA1c determined by direct measurement of patients’ blood) versus online collection (both zBMI and HbA1c calculated on the basis of parent-reported values) may have impaired the accuracy of those measurements. These issues of measurement validity lead us to view the changes in zBMI and the unchanged HbA1c values with caution.

In terms of DEB predictors, regression analyses revealed the associations between DEBs (i.e., dieting behavior), higher zBMI [17, 19, 67–69], and duration of illness [45], as reported by previous studies on youths with T1D.

In both groups, a key role seemed to be played by gender and age. In line with gender-related prevalence—both in the general adolescent population [70, 71] and in the T1D adolescent population [56, 69, 72]—girls showed higher DEBs than boys (as revealed by the majority of EAT subscales), regardless of illness and of age. In both groups, gender emerged as a significant predictor of general DEB attitudes and dieting behaviors. Similarly, age was found to be a significant predictor of DEB symptoms, revealing that adolescents with and without T1D showed higher levels of dieting behaviors—but lower attitude to oral control—than children (as revealed by adolescents’ lower Oral Control mean scores than children).

In line with evidence indicating adolescence as the developmental period during which eating disturbances typically emerge [73] and describing adolescents as frequently engaging in DEBs—both in general [74] and in the T1D population [19–21]—this data confirms that DEBs, especially in terms of dietary restrictions and low oral control, are primarily an adolescent problem. The absence of significant differences between patients and controls in eating problems leads to the consideration of DEBs more as an adolescent developmental issue rather than as a result of the challenges of living with a chronic illness. It is widely recognized that the rapid and dynamic cognitive, developmental, and emotional changes of adolescence (increased independence in decision making, turning to peer group for validation, wishing to be “fit”) combine with weight and body image concerns (facing the impact of body changes, giving more importance to body image, putting more focus and energy into searching for acceptance by peers, etc.). These are major issues that adolescents have to face that can favor the adoption of a variety of inappropriate and risk-taking behaviors, such as unhealthy weight control practices and habits [75, 76].

This study has several limitations. The major limitation is that data collection of the data relied on voluntary participation; thus, generalizing to a general T1D population should be done with caution. Additionally, the use of self-reported measures administered online allowed us to overcome the impossibility of conducting a traditional paper survey; however, such a data collection method may yield imprecise ratings of specific anthropometric/clinical data (e.g., height, weight, and blood glucose levels as reported by parents) and of subjective perceptions of behaviors, thoughts, and feelings that might not have been sincerely, accurately, or fully revealed. While a web-based survey can be an effective method of gathering data with evident advantages (easy to complete, quick collection of data, potentially lower costs, reduced survey administration overhead), incompatibility with the target environment (e.g., due to the closeness with parents or relatives), computer/smartphone literacy of participants, and program/app defects might negatively impact the quality of data collection [77]. Furthermore, given the cross-sectional nature of the results, longitudinal research is needed to further explore the relationship between DEBs and clinical and sociodemographic variables. In particular, given that it is essential to identify insulin misuse in addition to common DEBs in early detection and related interventions in order to reduce the risk of acute and late diabetes complications, future investigations will have to properly and accurately assess purging behaviors that are unique to individuals with T1D.

In conclusion, our findings suggest that, as recommended by international guidelines [12, 64, 78], continuous medical and psychological care is needed in order to periodically monitor physical and psychological conditions, especially during critical developmental phases, as teens age and/or face troubling circumstances such as those imposed by quarantine.

## Data Availability

The datasets used and/or analyzed during the current study are available from the corresponding author on reasonable request.
